# More ethical and more efficient clinical research: multiplex trial design

**DOI:** 10.1186/1756-0500-7-530

**Published:** 2014-08-14

**Authors:** Frederik Keus, Iwan CC van der Horst, Maarten W Nijsten

**Affiliations:** Department of Critical Care, University of Groningen, University Medical Center Groningen, Groningen, the Netherlands

**Keywords:** Trial design, Clinical equipoise, Factorial design, Simultaneous randomization, Interaction, Bias

## Abstract

**Background:**

Today’s clinical research faces challenges such as a lack of clinical equipoise between treatment arms, reluctance in randomizing for multiple treatments simultaneously, inability to address interactions and increasingly restricted resources. Furthermore, many trials are biased by extensive exclusion criteria, relatively small sample size and less appropriate outcome measures.

**Findings:**

We propose a ‘Multiplex’ trial design that preserves clinical equipoise with a continuous and factorial trial design that will also result in more efficient use of resources. This multiplex design accommodates subtrials with appropriate choice of treatment arms within each subtrial. Clinical equipoise should increase consent rates while the factorial design is the best way to identify interactions.

**Conclusion:**

The multiplex design may evolve naturally from today’s research limitations and challenges, while principal objections seem absent. However this new design poses important infrastructural, organisational and psychological challenges that need in depth consideration.

## Background

Randomised clinical trials (RCT) should provide high-level evidence. However RCT’s often exhibit several important limitations: (A) Clinical equipoise is frequently not present for the interventions that are compared [1]. (B) Patients are often not allowed to participate in multiple trials for mainly non-methodological reasons. (C) The costs to initiate, conduct and successfully conclude trials are high. (D) Trials may only include small proportions of the relevant patients leading to results with low external validity. (E) Frequently, trialists choose surrogate or intermediate outcomes [2, 3]. (F) And unrealistic, small sample sizes may be based on inappropriate optimism regarding the expected effect size [3]. All these factors can increase bias and can thus lead to unreliable conclusions [4].

Multiplex trial design may offer benefits including a more ethical and more efficient clinical research methodology.

## Findings

**Figure 1:**
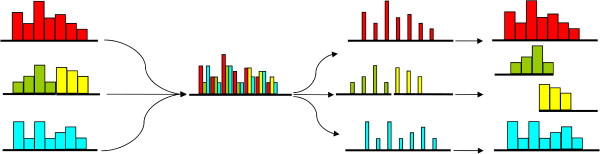
**Multiplex communication principle.** In communications technology and in other fields multiplexing is used to combine multiple independent packages of information into a continuous single signal that after transmission can be decomposed into the original information packages.

**Figure 2:**
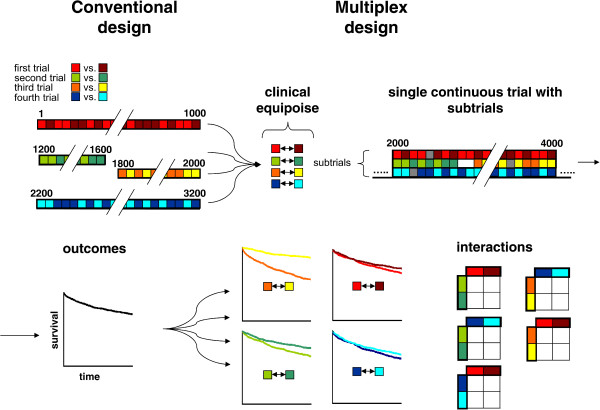
**Conventional versus multiplex trial design.** In most conventional randomized controlled trials, the effect of only one intervention is examined. Multiple questions are typically addressed in a serial manner. Thus in the first trial patients 1-1000 are studied to compare red and brown treatments, in the second trial patients 1200 -1600 (green vs. dark green) the third trial patients 1800-2000 (yellow vs. orange) and in the fourth trial patients 2200-3200 (light blue vs. dark blue). The different numbers of included patients result from different power requirements. The gaps in included patients are related with logistical issues. Within the multiplex trial concept as many questions as possible are addressed simultaneously, provided clinical equipoise exists between all treatments (and their combinations) that are examined. For example the three different subtrials may examine the two interventions for blood pressure, pain and fever respectively in patient 2000 through 4000. Although the subtrials use more patients and thus have greater power, a lower overall number of patients is required, underscoring the efficiency of factorial trial design. The continuous nature of the multiplex design also reduces “down-time” (as indicated by the breaks between the four convential trials). Interactions between treatments can only reliably be detected with a factorial design, although the needed sample size to do so requires advanced analysis.

We hypothesize that a novel design, called multiplex, will allow a more ethical and more efficient framework for conducting clinical studies. The principle of multiplexing [5] is widely used in many technical areas. Briefly, it denotes the combination of multiple signals into a single combined continuous signal that later can be decomposed into its individual signals (Figure 1). Clinical trial design could evolve into a similar approach (Figure 2).

The proposed multiplex trial has several key design elements, including a combination of the continuous and factorial approaches (Table 1). Patients are enrolled within a permanent trial structure and at any time point several subtrials will be conducted to address different hypotheses. The multiplex principle includes a factorial design to simultaneously run various subtrials for the same patient category [6, 7]. The number of clinical comparisons that are studied will vary over time, depending on the subtrials that are running at any time point. For all subtrials a relevant outcome according to Grading of Recommendations Assessment Development and Evaluation (GRADE) [2] is used. For example in critical care many interventions could be assessed for the agreed-upon outcome, i.e. hospital mortality. Enforcing clinical equipoise between the interventions within each subtrial, and their various combinations justifies that patients are simultaneously included in multiple subtrials. Such a design will result in increased efficiency as patients are randomised for multiple subtrials simultaneously and because of the continuous nature. It may demonstrate robust detection of interactions, increased patient consent by respecting equipoise and increased generalisability of results by using fewer exclusion criteria (Table 1).

**Table 1:** **Key elements, requirements and additional benefits of multiplex trial design**

Key elements	
A Clinical equipoise between treatment arms	
B Factorial design allows simultaneous participation in multiple subtrials	
C Continuous design so subtrials are embedded in a permanent infrastructure	
D Broad inclusion criteria	
E Relevant and robust outcome measures according to GRADE	
F Large sample sizes	
**Requirements**	
Treatment arms reflect current practice (A)	
Increased involvement of patients (B)	
Streamlined consent procedure for multiple subtrials (B)	
Multiple principal investigators must closely collaborate (B)	
Mutual acceptance of multiple sponsors (B)	
Extensive involvement of institutional review board (A,B,C)	
Integration with existing outcome registries (C,E,F)	
Advanced ICT infrastructure (B,C)	
**Benefits**	
Patients	More confidence in clinical research (A,B,E)
Scientific	Detection of interactions (B)
	Results with low risks of bias (A,D,E,F)
	Results with low risks of random error (A,D,E,F)
	Higher external validity (C,D,F)
Societal	More answers to relevant clinical questions (B,C,D,E,F)
	More efficient use of resources (B,C,D)

Summary of the components of the multiplex concept (A through F) and the various requirements as well as further benefits that we foresee that are related with these components.

### Key elements of multiplex design

(A) The condition of clinical equipoise is often not met by the interventions that are compared [8], for example because of an inappropriate control arm [9]. Freedman [1] proposed that ‘clinical equipoise’ should be based on present or imminent controversy in the clinical community over which treatment would be the preferred treatment. Thus this requirement is satisfied if there is genuine uncertainty within the expert medical community - not necessarily on the part of the individual investigator - about the preferred treatment [1]. For example, clinicians could be evenly divided whether epinephrine or norepinephrine would be optimal for a specific condition.

Despite strong debates, the universal nature of this ethical bedrock of trials cannot be ignored [8, 10]. Respecting clinical equipoise may well result in smaller expected differences in outcome. Consequently, the sample size estimation would be larger to adequately address the question of the subtrial.

(B) Patients do usually not participate in more than one trial. Competitive motives, more than scientific or patients’ interests, may explain this phenomenon. A patient who participates in several subtrials at the same time will contribute to several clinical questions in parallel and thus the costs per subtrial will decrease. In real life, patients who receive *N* different simultaneous treatments are thus subjected to a multitude, i.e. *N · (N-1)* of potential interactions. Interactions may remain undetected after approval and market launch [11, 12]. If possible, interactions ought to be identified during a trial, rather than many years after introduction of drugs or interventions into clinical practice. In case that a certain combination of treatments from different subtrials is known *a priori* to be undesired, such a particular combination could be excluded in the multiplex design.

(C) Currently the planning, initiation, conduct, conclusion and analysis of individual trials are associated with large costs. In contrast, the multiplex organisational and logistical structure - because of its permanent nature - does not need to be rebuilt once it has been established. Conducting randomisation superimposed on an existing registry was demonstrated to result in an important reduction in costs [13].

(D) Regarding inclusion criteria, many trials only include a small proportion of all eligible patients, when reported. Extensive use of exclusion criteria creates trial results applicable only for selected cases. Trials need to produce results which can be generalized to clinical practice by adopting wide inclusion criteria, and limited exclusion criteria [14]. Included patients need to represent the large majority of eligible patients. The multiplex trial design adopts wide inclusion criteria and as few exclusion criteria as possible. Within this framework, subgroup analyses may eventually reveal hypotheses for more or less favourable effects in specific patient groups.

(E) Concerning outcome, less important measures such as continuous outcomes and surrogate outcomes are frequently used, something mainly based on doctors’ or hospital’s interests (e.g. costs or hospital stay). GRADE emphasizes that outcome measures need to be ranked from to the perspective of the patient [2]. The multiplex trial design was conceived according to this principle: simple and robust outcomes such as mortality and (severe) adverse events that are most relevant to patients.

(F) Unfortunately, many trial sizes are too small to detect relevant statistical differences. Unwarranted optimism concerning intervention effects or the use of inappropriate surrogate outcomes are sometimes employed to justify small sample sizes. This may lead to false positive or false negative conclusions. There is considerable evidence that the majority of trials are underpowered, so definitive conclusions cannot be drawn for many clinical questions [15]. Usually far more randomised patients are needed before clinical questions can reliably be answered, and even more so when truly equivalent interventions are compared since differences will be smaller. Multiplex trial design therefore must entail large(r) sample sizes [16].

## Discussion

The combination of a continuous and factorial trial with clinical equipoise between all interventions, requires that each subtrial should be designed with low risks of bias and sufficiently large sample sizes based on realistic intervention effects focusing on outcome measures according to GRADE [2, 4, 15, 17].

Key elements of the multiplex design interact: both equipoise and choosing relevant outcomes inevitably lead to larger sample sizes as hypothesized differences in intervention effects will be smaller. This effect, however, will be offset by higher efficiency due to simultaneous randomisation of patients in multiple subtrials. A critical multiplex advantage is that only factorial studies can detect interactions [7, 18]. Since equipoise and quality are central principles of the proposed multiplex design, we believe that patient’s confidence and consent will increase.

Organisational issues as well as the willingness of investigators to cooperate must be addressed if multiplex is to succeed. Informed consent for multiple subtrials must be condensed into a single transparent informed consent procedure. Attitudes toward intellectual and material ownership of trials need to change fundamentally. Funding of multiplex trial units may originate from sponsors such as health service providers, drug and device industries, as well as clinical investigators. Although sponsors will have to relinquish part of their control of trials [19], in return the required resources would be borne by multiple sponsors. Ownership of trials as well as which subtrials are prioritized should be reconsidered as well as who will manage a multiplex institute. We believe that many registries (e.g. for cardiology or critical care) that currently record outcome on a nation-wide basis might provide part of the infrastructure for conducting multiplex trials. Modern information technology and web-based applications may offer solutions for data registration as well as transparency towards patients. Large sample sizes inevitably demand a multi-centre approach and probably the organisational structure will expand beyond national boundaries, creating additional legal challenges. Although these and other yet unthought-of issues must all be addressed before we can embark upon such a complex enterprise, we firmly believe that the multiplex trial design is a natural direction trial design must evolve into.

## Conclusion

We believe that the multiplex design is compatible with basic ethical principles and that it is a natural methodological direction that trial design should evolve into. Obviously, considerable psychological, organizational and regulatory hurdles have to be overcome before multiplex trial design becomes reality.
